# Broom versus broom-and-brush: A comparison of Surepath^®^ liquid-based Papanicolaou test (LBPT) collection devices

**DOI:** 10.4103/1742-6413.56360

**Published:** 2009-10-09

**Authors:** Min Yi Ngae, Clinton D Crowder, Klint Kjeldahl, Roberto Gamez, Samantha Paulson, Daniel M McKeon, Priti Goyal, Stefan E Pambuccian

**Affiliations:** Department of Laboratory Medicine and Pathology, University of Minnesota, Minneapolis, MN, USA; 1 University of Minnesota Medical Center, Fairview, Minneapolis, MN, USA

To the Editor,

We read with interest the recent *Cytojournal* article “Collection of the BD SurePath Pap Test with a broom device plus endocervical brush improves disease detection when compared to the broom device alone or the spatula plus endocervical brush combination” by Davis-Devine *et al.*,[1] in which the authors conclude that a combination of broom and brush is superior to broom alone as collection devices for Surepath^®^ Pap tests, increasing the endocervical cell recovery and the yield of low-grade squamous lesions (LSIL). We wish to report our findings from a similar study we conducted as they are somewhat different from those of Davis-Devine *et al.*[1] and may be of interest to the readers of *Cytojournal*. Preliminary findings from our study were presented at the United States and Canadian Academy of Pathology 2008 Annual Meeting.[2]

In our study, we specifically attempted to answer the question of whether the endocervical (EC) brush (Rovers^®^ EndoCervex-Brush^®^) (“brush”) demonstrated any additional utility when used in combination with the Rovers^®^ Cervex^®^ brush (“broom”), with respect to endocervical cell recovery or diagnostic rate in BD Surepath^®^ Pap tests.

A total of 14,147 BD Surepath^®^ vials were visually inspected in two randomly selected study periods (April 27, 2007 to July 2, 2007 and September 14, 2007 to December 28, 2007) and the presence and type of sampling devices were recorded. A total of 12,089 vials contained the broom alone, while 1,897 had a broom + brush combination. In addition, 125 vials contained only a brush and 36 contained no sampling device.

We then correlated the presence of a broom only or broom + brush combination with patient age, rate of EC cell recovery, and diagnostic rate [Table 1] and rate of each particular Bethesda 2001 diagnostic category [Table 2].

**Table 1:** Characteristics of broom versus broom + brush groups

*Sampling device*	*Number of specimens*	*Mean age*	*EC/TZ component present*	*Abnormal diagnoses*	*Unsatisfactory*
Broom alone	12,089	39.8	8,515 (70.6)	901 (7.5)	26 (0.2)
Broom + brush	1,897	42.6	1,513 (79.8)	132 (7.0)	2 (0.1)
Broom vs. broom + brush		<0.001	<0.0001	0.47	0.47
*P*-value					

EC/TZ = endocervical cell/transformation zone, Figures in parenthesis are in percentage

**Table 2:** Rate of each Bethesda 2001 diagnostic category

*Sampling device*	*ASC-US*	*LSIL*	*ASC-H*	*LSIL-H*	*HSIL*	*AGC*	*AdCa*
Broom alone	515 (4.3)	249 (2.1)	46 (0.4)	22 (0.2)	39 (0.3)	27 (0.2)	3 (0.02)
Broom + brush	80 (4.2)	32 (1.7)	10 (0.5)	3 (0.2)	5 (0.3)	2 (0.1)	0 (0)
Broom vs. broom + brush	0.98	0.32	0.46	0.82	0.84	0.44	0.49
*P*-value							

ASC-US: Atypical squamous cells of undetermined significance; LSIL: Low grade squamous intraepithelial lesion; ASC-H: Atypical squamous cells, Cannot Exclude HSIL; LSIL-H: Low grade squamous intraepithelial lesion, Cannot Exclude HSIL; HSIL: High-grade squamous intraepithelial lesion; AGC: Atypical glandular cells; AdCa: Adenocarcinoma, Figures in parenthesis are in percentage

We found that the broom + brush combination was used to sample older women, which may indicate that clinicians who submit specimens to our laboratory may be preferentially using the EC brush to ensure sampling of the transformation zone (TZ) in older women. Despite the fact that it was used in older women, the broom + brush combination ensured a higher rate of EC cell recovery than the broom alone.

We found no significant differences in the overall detection rate of abnormalities or for any diagnostic category between the Pap tests collected with the broom + brush combination and those collected with the broom only. However, since women sampled with the broom + brush combination were significantly older than those sampled with the broom alone, we had expected a lower abnormal rate in the broom + brush combination group based on the documented decline in cervical abnormalities with increasing age.

In trying to elucidate the reasons why we did not find a higher abnormal or a higher LSIL detection rate while Davis-Devine *et al*.[1] did, we considered two possible explanations. Our study was slightly smaller in size with 12,089 broom and 1,897 broom + brush cases, as compared to 11,130 broom and 2,921 broom + brush cases in their study, but we believe that this difference did not significantly impact our respective results. It is more likely that the differences in the results between the two studies are related to differences in the two patient populations undergoing screening.

The population in the study by Davis-Devine *et al*.[1] had a higher calculated abnormal rate (10.5% vs. 7.4% in our study), largely due their higher rates of ASC-US (6.4% vs. 4.3%) and LSIL (3.1% vs. 2%), while their HSIL rate was lower (0.23% vs. 0.31%).

Abnormal Pap test rates vary considerably in the literature, likely reflecting both the potential variability in cytologic diagnoses between institutions and a combination of different patient population characteristics such as age, location, socioeconomic status, prevalence of HPV, and HIV infection as well as initial and continued access to adequate preventive healthcare.

Davis-Devine *et al*.[1] do not provide age data for their population, however, based on the higher rate of both ASC-US and LSIL we suspect that their study population may be younger than ours.

Like Davis-Devine *et al.*, our study was probably underpowered for comparisons in detection rates of HSIL and more severe diagnoses.

In conclusion, the results of our study support the idea that a better sampling of the EC/TZ can be achieved with the broom + brush combination as compared to sampling with the broom alone.

However, we believe that additional larger studies on the subject of collection devices, including the newer and potentially easier to use Rovers^®^ Cervex-Brush^®^ Combi (which combines the broom and the brush into one device) in the setting of liquid-based Pap testing are needed. Such studies need to allow age-controlled comparisons and correlation with human papillomavirus DNA test status before we can conclude that sampling with any device or combination of devices allows the detection of a higher rate of significant cervical abnormalities.

## COMPETING INTEREST STATEMENT BY ALL AUTHORS

No competing interest to declare by any of the authors.

## AUTHORSHIP STATEMENT BY ALL AUTHORS

All authors of this article declare that we qualify for authorship as defined by ICMJE http://www.icmje.org/#author.

Each author has participated sufficiently in the work and take public responsibility for appropriate portions of the content of this article.

Each author acknowledges that this final version was read and approved.

## ETHICS STATEMENT BY ALL AUTHORS

This study was conducted with approval from Institutional Review Board (IRB) (or its equivalent) of all the institutions associated with this study. Authors take responsibility to maintain relevant documentation in this respect.
